# Mycoplasma pneumonia infection and asthma: A clinical study

**DOI:** 10.12669/pjms.313.7042

**Published:** 2015

**Authors:** Shuncui Gao, Lingcheng Wang, Wei Zhu, Jie Jiang

**Affiliations:** 1 Shuncui Gao, Department of Respiratory Medicine, Fourth People’s Hospital of Jinan City Shandong Province, 250031, China; 2 Lingcheng Wang, Department of Respiratory Medicine, Fourth People’s Hospital of Jinan City Shandong Province, 250031, China; 3 Wei Zhu, Department of Respiratory Medicine, Fourth People’s Hospital of Jinan City Shandong Province, 250031, China; 4 Jie Jiang, Department of Respiratory Medicine, Fourth People’s Hospital of Jinan City Shandong Province, 250031, China

**Keywords:** Mycoplasma pneumonia infection, Pulmonary function tests, Sputum induction examination, IgE in serum, Asthma

## Abstract

**Objectives:**

To evaluate the correlation between mycoplasma pneumonia infection and the severity of asthma as well as asthma control, to help physicians in respiratory department better make treatment strategies.

**Methods:**

Since January 2012 to May 2014, we consecutively recruited 149 out-patients diagnosed with asthma in acute or convalescent phase from the department of respiratory medicine of our hospital. The pulmonary function tests, sputum induction examination, measurement of IgM, IgG and IgE in serum, evaluation of asthma control were carried out for all the included patients.

**Results:**

In 78 cases with asthma in acute phase, mycoplasma pneumonia infection was confirmed in 38 cases (48.71%), and in 71 cases in stable state, mycoplasma pneumonia infection was confirmed in 22 cases (30.98%). There was significant difference in the rate of mycoplasma pneumonia infection between the two groups (p<0.05). The FEV1% Pred and ACT scores were significantly lower in mycoplasma pneumonia infection cases than those in no mycoplasma pneumonia infection cases (p<0.05), while the eosinophil count and IgE in serum were significantly higher in mycoplasma pneumonia infection cases (p<0.05).

**Conclusions:**

Mycoplasma pneumonia infection may play more important role in the occurrence of acute asthma, and it can lead to decreased pulmonary function, difficulty in controlling asthma and more severe airway inflammation.

## INTRODUCTION

Asthma is a chronic inflammatory disease of airways characterised by airways hyper-responsiveness to a multiplicity of stimuli and the pathogenesis may be the result of a complicated factors including genetic and environmental influences.^1^ The disease represents an important healthcare problem, accounting for a high rate of morbidity and mortality, and has been paid a high attention by physicians in respiratory department.^2^

Respiratory infections are one of the major causes of asthma exacerbations.^3^ The role of respiratory tract infections caused by mycoplasma pneumonia in the pathogenesis of asthma has been well established and physicians suggested mycoplasma pneumonia infection seems to be important in asthma pathogenesis and the clinical course of the disease.^4^ In terms of the exact influence of mycoplasma pneumonia on asthma, some authors suggested that mycoplasma pneumonia infection can precede the onset of asthma, exacerbate asthmatic symptoms, and cause difficulties with asthma management.^5^,^6^ On the other hand, some authors found that the asthma control status and parameters of lung function tests did not differ between asthmatic patients with and without evidence of mycoplasma pneumonia infection, and advocated that mycoplasma pneumonia infection is not related to asthma control, asthma severity, and location of airway obstruction.^7^ Consequently, the correlation between mycoplasma pneumonia infection and the degree of severity of asthma as well as asthma control has not been yet evaluated clearly.

Therefore, we carried out a clinical study prospectively, using 149 cases with acute or chronic asthma, between Jan 2012 and May 2014. Our objectives were: 1) To evaluate a possible correlation between mycoplasma pneumonia infection and the severity of manifestation of asthma as well as asthma control; 2) To help physicians in respiratory department better make treatment strategies.

## METHODS

This study was designed and carried out in the department of respiratory medicine, fourth People’s Hospital of Jinan City, Shandong province, China, between Jan 2012 and May 2014. In the current study, we consecutively recruited 149 out-patients who were diagnosed with asthma in acute or convalescent phase from the department of respiratory medicine of our hospital. In 149 patients, 78 were asthma in acute phase and 71 in convalescent phase. Among 78 acute cases, 43 were male and 35 female with an age ranged from 41 to 54 years old; among 71 convalescent cases, 34 were male and 37 female with an age ranged from 43 to 56 years old. There were no significant difference in the basic clinical data such as age, gender and medicine history between the two groups.

Inclusion criteria of the current study were doctor-diagnosed asthma, with or without signs or symptoms of an asthma exacerbation (cough, dyspnea, wheezing and/or chest pain), need or ever need for treatment with systemic corticosteroid and inhaled bronchodilator as determined by the attending physician, and availability for phone follow up.^8^ The study was approved by the ethical committee of our hospital and carried out according to the principles of Helsinki Declaration. All the included cases gave informed consent.

### Procedures

The following procedures were carried out in the two groups. (1) For each included patient, 2 ml fasting venous blood was drawn for the detection of specific IgM and IgG, for which the EIA-Platelia was performed manually according to the manufacturer’s recommendations. The test result was validated as instructed in the kit manual.^9^ (2) The test of pulmonary function including the forced vital capacity (FVC), forced expiration volume of one second (FEV_1_), peak forced expiratory flow (PEF), and the percentage of forced expiration volume of one second from the predicted value (FEV_1_% pred) were measured using cardiopulmonary measuring instruments.^10^ The test was done three times and the averages of the measured levels were used in the study.^10^ (3) The level of asthma control in each patient was assessed by Asthma Control Test (ACT).^11^ (4) Sputum induction examination were performed according to the previous literatures.^12^,^13^ The patients received 200µg of salbutamol before the induction. After the post bronchodilator spirometry they inhaled sterile hypertonic saline at increasing concentrations at room temperature via a pneumatic nebulizer with the output set at 0.35 ml/min. The duration of each inhalation was 5 minutes and the induction was stopped after expectoration of an adequate amount of sputum.^14^ Induced sputum was analyzed immediately and the cell counting of eosinophil was performed.(5) Content of IgE in serum was measured using the ImmunoCAP system.^15^

### Statistical analysis

Statistical analysis was performed using SPSS17.0 (Chicago, IL, USA). The comparison of measurement data between two groups was performed using independent 2-sample t test. The comparison of enumeration data between two groups was carried out using Chi-Square test. The correlation between mycoplasma pneumonia infection, ACT scores, FEV1% pred, and content of IgE in serum were performed using Pearson correlation analysis. A p-value less than 0.05 was considered to indicate statistical significance.

## RESULTS

In 78 cases with asthma in acute phase, mycoplasma pneumonia infection was confirmed in 38 cases (48.71%), and in 71 cases with asthma in stable state, mycoplasma pneumonia infection was confirmed in 22 cases (30.98%). There was significant difference in the rate of mycoplasma pneumonia infection between the two groups (p<0.05).

The comparison of pulmonary function, eosinophil count in sputum induction examination and content of IgE in serum between mycoplasma pneumonia infection and no mycoplasma pneumonia infection cases in the group of acute asthma were listed in Table-I. The FEV1%pred and ACT scores were significantly lower in mycoplasma pneumonia infection cases than those in no mycoplasma pneumonia infection cases (p<0.05), while the eosinophil count and IgE in serum were significantly higher in mycoplasma pneumonia infection cases (p<0.05) (Table-I).

**Table-I:** Comparison between cases with or without mycoplasma pneumonia infection in acute asthma group.

	FEV_1_%pred (%)	ACT score	Eosinophil count (%)	IgE in serum (U/L)
MP infection(38)	62.1±9.7	18.5±2.1	32.2±18.6	330.1±29.7
No MP infection (40)	71.5±10.1	21.7±2.4	17.4±12.8	269.4±36.5
P value	<0.05	<0.05	<0.05	<0.05

*NOTE:* MP=mycoplasma pneumoniae

In cases with asthma in stable state, the comparison of related parameters were listed in Table-II. The mycoplasma pneumonia infection cases have relatively lower ACT scores (p<0.05), but higher in Ig E concentration and eosinophil count (p<0.05), when compared with the cases without mycoplasma pneumonia infection (Table-II).

**Table-II:** Comparison between the cases with or without mycoplasma pneumonia infection in stable asthma group.

	FEV1%pred (%)	ACT score	Eosinophil count (%)	Ig E in serum (U/L)
MP infection(22)	73.8±9.8	20.2±1.9	9.7±3.6	231.7±23.9
No MP infection (49)	78.2±11.3	22.6±2.3	7.8±3.2	198.4±24.5
P value	<0.05	<0.05	<0.05	<0.05

*NOTE:* MP= mycoplasma pneumonia

In addition, we found in the current study, ACT score is positively correlated (p<0.05) to FEV1%pred, and negatively correlated (p<0.05) to the percentage of the Eosinophil count, but no significant correlation between other parameters (P>0.05).

## DISCUSSION

A number of respiratory infections have been reported to be involved in the occurrence of asthma, in which mycoplasma pneumonia is an organism having a strong relationship to asthma and reported by many authors.^4^,^7^ In the current study, we found that the rate of mycoplasma pneumonia infection was 48.71%in acute asthma group, and 30.98%in convalescent group, demonstrating that the percentage of mycoplasma pneumonia infection in asthma patients was high, which confirmed the previous viewpoints.^4^,^7^ At the same time, the infection rate in convalescent group is significantly lower than that in acute asthma group, indicating that the mycoplasma pneumonia infection may play more important role in the occurrence of acute asthma, which is consistant with the points resulted from Zhou’s study.^16^

Moreover, in a study of sixty-two patients, Khalil and colleagues revealed that the ACT scores and parameters of lung function tests did not differ between asthmatic patients with and without evidence of chronic mycoplasma pneumonia infection.^7^ However, we found in both acute and convalescent group, the values of FEV_1_%pred and ACT scores were significantly lower, but the eosinophil count in sputum induction examination and IgE in serum were significantly higher in mycoplasma pneumonia infection patients, than those in no-mycoplasma pneumonia infection patients. In the field of asthma, ACT scores was carried out usually to assess the control of asthma, and eosinophil count in sputum induction examination to evaluate the level of airway inflammation. The current results may indicate that the mycoplasma pneumonia infection can lead to decreased pulmonary function, difficulty in controlling asthma and more severe airway inflammation. In terms of the difference between the current study and Khalil’s study, it may be attributed to the race, region, accuracy of test or many other factors, which may need further study to clarify.

Also, we found in the current study, ACT score is positively correlated to FEV_1_%pred, and negatively correlated to the percentage of the eosinophil count in sputum induction examination, which indicated that even in patients with asthma in convalescent phase, there is inflammation resulted from eosinophil available, and lower control level of asthma was correlated with higher inflammation level. In addition, we found in the current study the mycoplasma pneumonia infection seems to be correlated to FEV_1_%pred, ACT scores, eosinophil count in sputum induction examination and IgE in serum positively or negatively, but we didn’t find significant difference in statistical analysis, while we suggest that there is such a trend available.

## References

[1] Nisar N, Guleria R, Kumar S, Chand Chawla T, Ranjan Biswas N (2007). Mycoplasma pneumonia and its role in asthma. Postgrad Med J.

[2] Cosentini R, Tarsia P, Canetta C, Graziadei G, Brambilla AM, Aliberti S (2008). Severe asthma exacerbation: role of acute Chlamydophila pneumoniae and Mycoplasma pneumonia infection. Respir Res.

[3] Specjalski K (2010). Role of Chlamydia pneumoniae and Mycoplasma pneumonia infections in the course of asthma. Pneumonol Alergol Pol.

[4] Blanchard E, Raherison C (2010). Asthma and Mycoplasma pneumonia. Rev Mal Respir.

[5] Hong SJ (2012). The Role of Mycoplasma pneumonia Infection in Asthma. Allergy Asthma Immunol Res.

[6] MacDowell AL, Bacharier LB (2005). Infectious triggers of asthma. Immunol Allergy Clin North Am.

[7] Ansarin K, Abedi S, Ghotaslou R, Soroush MH, Ghabili K, Chapman KR (2011). Infection with Mycoplasma pneumonia is not related to asthma control, asthma severity, and location of airway obstruction. Int J Gen Med.

[8] Arnold DH, Gebretsadik T, Abramo TJ, Hartert TV (2012). Noninvasive testing of lung function and inflammation in pediatric patients with acute asthma exacerbations. J Asthma.

[9] Sobieszczanska BM, Kasprzykowska U, Duda-Madej A, Secewicz A, Marciniak J, Gosciniak G (2014). Relevance of serology for Mycoplasma pneumonia infection among children with persistent cough. Adv Clin Exp Med.

[10] Roh H, Lee D (2014). Respiratory function of university students living at high altitude. J Phys Ther Sci.

[11] Emami M, Tayebi A, Gharipour M, Farzamnia S, Temyarti AK (2014). Comparing clinical efficacy of Symbicort versus Pulmicort in reducing asthma symptom and improving its control. Adv Biomed Res.

[12] Demedts IK, Morel-Montero A, Lebecque S, Pacheco Y, Cataldo D, Joos GF (2006). Elevated MMP-12 protein levels in induced sputum from patients with COPD. Thorax.

[13] Van Pottelberge GR, Mestdagh P, Bracke KR, Thas O, van Durme YM, Joos GF (2011). Micro RNA expression in induced sputum of smokers and patients with chronic obstructive pulmonary disease. Am J Respir Crit Care Med.

[14] Domagala-Kulawik J, Maskey-Warzechowska M, Hermanowicz-Salamon J, Chazan R (2006). Expression of macrophage surface markers in induced sputum of patients with chronic obstructive pulmonary disease. J Physiol Pharmacol.

[15] Liu JN, Shin YS, Yoo HS, Nam YH, Jin HJ, Ye YM (2014). The Prevalence of Serum Specific IgE to Superantigens in Asthma and Allergic Rhinitis Patients. Allergy Asthma Immunol Res.

[16] Zhou A, Dai Y, Shen X, Chen Z, Shen H (2014). Correlations of mycoplasma pneumonia infection with airway inflammation and asthma control in patients with bronchial asthma. Chinese J Pract Internal Med.

